# Effect of Bilateral Erector Spinae Plane Block on Postoperative Analgesia in Cesarean Section Under Spinal Anaesthesia: A Prospective Randomized Controlled Trial

**DOI:** 10.4274/TJAR.2024.241538

**Published:** 2024-07-12

**Authors:** Bengi Şafak, Onat Bermede, Süheyla Karadağ Erkoç, Volkan Baytaş, Bulut Varlı, Asuman Uysalel

**Affiliations:** 1 Ankara University Faculty of Medicine, Department of Anaesthesiology and Intensive Care, Ankara, Turkey; 2 Ankara University Faculty of Medicine, Department of Obstetrics and Gynecology, Ankara, Turkey

**Keywords:** Analgesia, cesarean section, nerve block, pain, pain measurement

## Abstract

**Objective:**

Acute pain after cesarean section (CS) can affect the quality of life of patients. This study aimed to assess the impact of bilateral erector spinae plane block (ESPB) under spinal anaesthesia on postoperative pain, analgesic usage, and patient satisfaction in elective CS.

**Methods:**

A total of 116 ASA II females aged 18-45 years who had elective CS were included in this prospective randomized study. Adjusted for the patient’s height and weight, 0.5% bupivacaine and 12.5 µg fentanyl were administered for spinal anaesthesia. In the ESPB group, ultrasonography-guided ESPB with 10 mL 0.5% bupivacaine+10 mL saline was applied bilaterally at the T12 vertebrae level at the end of the surgery. Postoperative analgesia was planned with diclofenac and paracetamol. Patients’ satisfaction, analgesic usage, rest, movement, cough, and low back pain were evaluated using a visual analogue scale (VAS) at postoperative hours 2, 4, 6, 12, and 24. The extent of the sensory block level of ESPB was evaluated after the spinal anaesthesia had worn off.

**Results:**

The analysis included 49 patients in the ESPB group and 50 in the control group with comparable demographics. Rest, movement, and cough VAS scores were substantially lower at the 2^nd^, 4^th^, 6^th^, and 12^th^ h in the ESPB group, and satisfaction was better. Total analgesic consumption and the need for rescue analgesics were higher in the control group. VAS scores and ESPB spread levels are negatively correlated.

**Conclusion:**

As a safe component of multimodal analgesia following CS, bilateral ESPB can be effectively performed.

**Main Points**

⦁ Cesarean section (CS) is associated with severe postoperative pain.

⦁ It is important to plan a multimodal analgesic treatment for post-CS pain considering both the mother’s and the infant’s quality of life.

⦁ Erector spinae plane block (ESPB) is an effective component of analgesic treatment for various types of surgical procedures.

⦁ The results of this study revealed that bilateral ESPB in patients delivered by elective CS under spinal anaesthesia can result in decreased visual analogue scale scores, prolonged time until the first analgesic request, decreased usage of rescue analgesics, and increased satisfaction.

## Introduction

Cesarean section (CS) is associated with severe postoperative pain.^1^ The diversity of acute pain intensity after CS makes pain intensity prediction difficult due to its variability. During the acute period after CS, discomfort might interfere with daily activities such as walking, emotions, sleep, communication, and concentration. Severe acute pain can lead to persistent postpartum pain and depression.^2^ Untreated post-CS pain can affect mother-child interaction and increase psychosocial and health problems in children.^3^ Post-CS pain is a combination of pain from the visceral organs, skin incision, and lower back. The effect of drugs on newborns affects the management of post-CS pain. In multimodal analgesia regimens, the combination of oral analgesic drugs with neuraxial opioids and regional nerve block techniques provides efficient analgesia and improves recovery outcomes.^4^

The erector spinae plane block (ESPB) is a clear example of an interfacial plane block.^5^ Since its initial description, it has been used for various clinical conditions.^6^ Drug dissemination to the multisegmental epidural and paravertebral spaces, ventral and dorsal rami, sympathetic chain, and intercostal space has been reported.^7^ Because it provides effective visceral and somatic analgesia,^8^ ESPB could be a beneficial regional block approach for managing post-CS pain. Because of physiological changes during pregnancy, fewer neuraxial anaesthetics are required. A regional nerve block technique and a low-dose neuraxial anaesthetic drug combination can enhance recovery. The effects of ESPB in obstetric anaesthesia have been reported in a limited number of randomized controlled trials.

Patient outcomes following elective CS under spinal anaesthesia were the focus of our investigation; the primary objective was to assess the impact of bilateral ESPB on postoperative pain as measured by the visual analogue scale (VAS). The secondary objectives were to assess the impact of bilateral ESPB on postoperative analgesic medication usage and patient satisfaction.

## Methods

**Figure 1:**
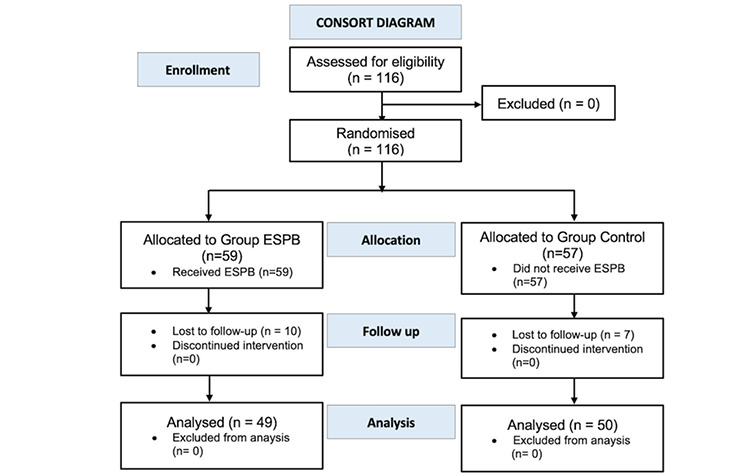
CONSORT flow diagram for the study

This prospective randomized study was conducted between May 2020 and June 2021 in a university hospital with the approval of the Human Research Ethics Committee of Ankara University Faculty of Medicine (date: 13.02.2020, approval no.: İ2-87-20). The study was registered at the Clinical Trials Protocol Registration and Results System (ClinicalTrials.gov ID: NCT05695625) and was conducted following the principles of the Declaration of Helsinki. Enrollment and allocation are shown in the CONSORT flow diagram (Figure 1). The American Society of Anesthesiologists (ASA) health status II female patients aged between 18 and 45 years who delivered by elective CS under spinal anaesthesia were included after waiving written informed consent. Patients under 18 years of age, over 45 years of age, ≥ASA health status III, who refused to participate, with body mass index >35 kg m^-2^, multiple pregnancy, preeclampsia, any contraindications for regional anaesthesia, known allergy to the study drugs, and a history of chronic substance or opioid use were excluded. Using the sealed envelope method, patients were randomly assigned to one of two groups: the ESPB group, which received bilateral ESPB at the end of surgery in addition to spinal anaesthesia; or the control group, which received spinal anaesthesia alone.

After standardized monitoring, intravenous (IV) access was achieved, and a balanced electrolyte solution was started. Under standard aseptic precautions, spinal anaesthesia was administered to all patients while they were seated at the lumbar 4-5 interspace. Following the observation of free cerebrospinal fluid flow, spinal anaesthesia was administered with 0.5% bupivacaine, adjusted for the patient’s height and weight (Table 1),^9^ and 12.5 g (µg) fentanyl. The patient was positioned 15° to the left, and 2 lt/min of oxygen was started via a nasal cannula. The administration time of spinal anaesthesia was recorded as the 0^th^ minute (min). The sensorial block was examined using a pinprick test, and the time required for the block to reach the T4 dermatome level and the Bromage score at that point were recorded. A 20% reduction in systolic blood pressure from baseline was defined as hypotension, and 100 µg of ephedrine was administered. A decrease in the heart rate below 60 beats per minute was defined as bradycardia, and 0.5 mg of atropine was administered.

**Figure 2:**
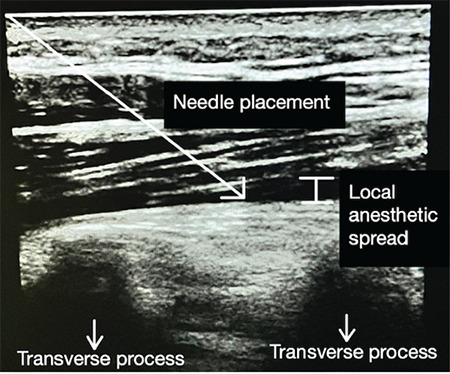
The USG image of the erector spinae plane block USG, ultrasonography.

In the ESPB group, two people assisted the patients into a sitting position at the end of the surgery. The spinous process of the T12 vertebra was confirmed by palpating cranially from the T7 vertebra and caudally from the L5 vertebra. After skin asepsis, a high-frequency linear ultrasonography (USG) probe (Samsung HM70A USG machine and Samsung L5-13IS USG probe, Korea) was placed in the midline. After visualizing the T12 vertebra spinous process, the probe was laterally moved approximately 4 cm to visualize the transverse process. Until the needle tip touched the transverse process, a 20 gauge 10 cm block needle (BRAUN Stimuplex Ultra 360, Germany) proceeded in the plane. The position of the needlepoint was verified by hydrodissection. Twenty milliliters of local anaesthetic solution (10 mL of 0.5% Bupivacaine +10 mL of 0.9% NaCI) was injected between the erector spinae muscle and the transverse process at a standard rate (Figure 2). The block was reproduced with equal volume and content on the opposite side of the back. The same anaesthesiologist performed all the blocks. The control group patients did not receive ESPB.

At the postoperative 2^nd^, 4^th^, 6^th^, 12^th^, and 24^th^ hours, all patients were visited in their rooms, and their rest, movement, cough, and low back pain VAS scores were evaluated (0: no pain- 10: worst pain). In case patients had a headache, it was planned to be evaluated with a VAS score. For postoperative analgesia, as a rescue analgesic, 75 mg of diclofenac (maximum dose 150 mg day^-1^) was intramuscularly administered to patients whose VAS score exceeded 4. After 30 min, patients with a VAS score greater than 4 received 1000 mg IV acetaminophen (maximum dose 4 gr day^-1^). Patients with severe pain were administered IV fentanyl through a patient-controlled analgesia system and were excluded from the study. Using a pinprick test at the midaxillary line and motor skills, the level of ESPB spread was evaluated as dermatomal 4-5 h after spinal anaesthesia had worn off.

The demographics of patients, amount of bupivacaine used for spinal anaesthesia, time of reaching the T4 level of the sensorial block, intraoperative hypotension and bradycardia, ephedrine and atropine requirement, operation time, ESPB application time, first mobilization time, analgesic consumption, first analgesic request time, patient satisfaction (0: very satisfied- 10: not satisfied), postdural puncture headache, breastfeeding, nausea and vomiting, and length of hospital stay were recorded.

### Statistical Analysis

The sample size was estimated with 80% power and a significance threshold of 0.05, assuming that a 1-unit change in the VAS value would be considered significant. It was calculated that at least 41 participants would be in each group, for a total of 82, and G*Power 3.1.9.2 was used for the calculation of samples.

The SPSS 11.5 program was used to analyze the data. Qualitative variables were represented by the number of patients stated as a percentage, while quantitative variables were described using the mean ± standard deviation. The presence of a difference was examined using the Mann-Whitney U test in the absence of normal distribution assumptions and the Student’s t-test in the presence of a distinction between categories of a qualitative variable and two categories of a quantitative variable. A chi-square test was used to investigate the correlation between the two qualitative variables. In the absence of adherence to the assumptions of normal distribution, the relationship between two quantitative variables was examined using Spearman’s correlation. *P* < 0.05 was determined as the statistical significance level.

## Results

One hundred and sixteen patients were included in the study despite the possibility of data loss. Ten patients in the ESPB group and seven in the control group with a lack of postoperative follow-up data were excluded from the statistical analysis. The statistical analysis included 49 patients in the ESPB group and 50 patients in the control group (Figure 1. CONSORT flow diagram). Demographics and intraoperative variables were comparable between the groups (Table 2).

The ESPB group had significantly reduced rest, movement, and cough VAS values in comparison to the control group during the 2^nd^, 4^th^, 6^th^, and 12^th^ h; nevertheless, no significant difference was observed between the groups by the 24^th^ h (Table 3). At all times, satisfaction was significantly better in the ESPB group (Table 3). In the ESPB group, the mean time until the first analgesic request was remarkably longer than that in the control group (5.03±4.99 hours vs. 2.49±1.21 hours, respectively, p<0.001). Total diclofenac consumption and the need for rescue analgesics in the early postoperative period were higher in the control group than in the ESPB group (128.80±56.27 mg vs.178.72±61.67 mg, *P *< 0.001 and 1.61±0.84 vs. 2.22±0.97, respectively, *P*=0.001). In the ESPB group, the mean number of acetaminophen administrations was 0.71±0.84 and it was 0.98±0.74 in the control group (*P*=0.047). None of the patients who participated in the study required fentanyl.

There was a weak negative correlation between the 4^th ^and 6^th^ hour VAS value for rest and the range of sensory block level (r=-0.293 and *P*=0.041, r=-0.298 and *P*=0.038, respectively). There was a moderately negative correlation between the 6^th^ and 12^th^ hour VAS value for movement and the range of sensory block level (r=-0.404 and *P*=0.004, r=-0.317 and *P*=0.027, respectively). There was a moderately negative correlation between the 6^th^ and 12^th^ hour VAS value for cough and the range of sensory block level (r=-0.426 and *P*=0.002, r=-0.302 and *P*=0.035, respectively).

The first mobilization time and length of hospital stay were similar between the groups (Table 2). All patients had no difficulty breastfeeding or caring for their infants. Neither nausea or vomiting nor headache was observed in any patient.

## Discussion

This study showed that bilateral ESPB in patients delivered by elective CS under spinal anaesthesia can result in decreased VAS scores, prolonged time until the first analgesic request, decreased usage of rescue analgesics, and increased satisfaction.

Regional anaesthesia is commonly preferred in elective CS because of the adverse effects of systemic drugs on newborns. For postoperative analgesia, the systemic use of opioids should be avoided because they pass into breast milk and may have adverse effects on the newborn.^10^ Intrathecal opioids can be added for longer postoperative analgesia; however, they can cause negative effects such as pruritus and respiratory depression.^11^ Because of these factors, the combination of regional and spinal anaesthesia has gained popularity. For numerous types of surgical procedures, ESPB has been confirmed to be effective as a part of multimodal analgesia. In a randomized controlled study by Hamed et al.^12^ comparing the use of ESPB and intrathecal morphine (ITM) in analgesic treatment after CS, bilateral ESPB was administered at the end of the operation in the ESPB group. During the postoperative period, the ITM group reported higher rest and cough VAS scores.^12^ The ESPB group showed significantly reduced rest and cough VAS scores compared with the control group in a randomized study conducted by Dostbil et al.^13^ Similarly, in our research, the ESPB group exhibited substantially reduced rest, movement, and cough VAS scores at the 2^nd^, 4^th^, 6^th^, and 12^th^ h. Regarding VAS scores, there was no significant difference between the groups at the 24 h evaluation. This may be because the impact of ESPB reduced after 24 h, and diclofenac and acetaminophen were used instead of opioids as rescue analgesics.

Pain after CS may be caused by the somatic fibers of the incision, uterine incision and contraction, and the peritoneum’s interaction with the uterus; therefore, analgesic activity is required to cover the thoracic, lumbar, and sacral nerve roots. ESPB stands out in post-abdominal surgery pain management because the dissemination of local anaesthetic is not limited to the injection level but can expand to the upper and lower vertebral levels. ESPB can extend to the sympathetic chain, dorsal and ventral ramus of the spinal nerves, and epidural and paravertebral areas.^7^ Therefore, the somatic and visceral components of abdominal innervation that originate from the lower thoracic levels can be blocked by ESPB.^8, 14^ Boules et al.^15^ and Malawat et al.^16^ compared the analgesic effects of transversus abdominis plane block (TAPB) and ESPB following CS, and VAS scores were lower in the ESPB groups. TAPB is effective for treating abdominal wall-related somatic pain with little or no visceral analgesia because of its impact on thoracolumbar nerves.^17^ Because of its impact on visceral nerves, ESPB provides more effective analgesia than TAPB.

ESPB spread may not proceed in living organisms, as in cadaveric studies; the amount of drug administered, active muscle tone, and intra-abdominal pressure may impact this spread. According to a study on the spread of local anaesthetics and cutaneous sensation loss following ESPB in volunteers, local anaesthetics consistently spread to the dorsal ramus, paravertebral region, and neural foramina, but epidural space spread was not always observed.^18^ The analgesic effects of unilateral ESPB can be bilateral.^19^ Due to the pneumoperitoneum and position in laparoscopic procedures, bilateral local anaesthetic spread may occur following unilateral ESPB.^20^ Unilateral ESPB applied via a catheter at the lumbar vertebrae level provided bilateral analgesia during labor.^21^ In the literature, a single shot bilateral ESPB with 20 mL solution caused sensory block at a mean of 7.36±0.9 dermatome levels, ranging from 6 to 9.^14^ ESPB spread is susceptible to variation based on the solution volume and location of administration.^7^ Although previous studies have noted an increase in the cephalocaudal spread with higher applied volume,^22^ the precise relationship between spread and volume remains obscure. Drug distribution observed because of ESPB is not always correlated with sensorial block.^18^ In our study, although the sensory extent of ESPB was recorded after the spinal anaesthesia had worn off, it may not have been correctly measured. It was observed that as the extent of the sensory block level expanded, patients’ VAS scores decreased. Considering these findings, it is necessary to conduct prospective research on this topic because additional variables may alter drug distribution. Consequently, the analgesic effectiveness of plane blocks can be revealed with greater clarity.

Our secondary aim was to evaluate the effects of bilateral ESPB on analgesic use and patient satisfaction during the postoperative period. Opioid usage was considerably reduced in the ESPB group compared with the control group in the study by Aygun et al.,^23^ evaluating the postoperative analgesic effects of USG-guided ESPB in post-CS patients. Likewise, we observed that the ESPB group consumed fewer analgesics. In a meta-analysis evaluating the effect of USG-guided ESPB following abdominal surgery, ESPB reduced opioid consumption in the first 24 h and prolonged the first analgesic usage time.^24^ Hamed et al.^12^ found that ESPB provided more long-lasting analgesia than intrathecal morphine. The mean time for the first analgesic usage was 12±2.81 hours in the ESPB group. In our study, the mean time for the first analgesic usage in the ESPB group was 5.03±4.99 hours. This may be the result of using fentanyl as our intrathecal opioid and administering nearly half the amount of bupivacaine used by Hamed et al.^12^ Increasing the amount of local anaesthetic used in ESPB may lead to more effective and long-lasting analgesia, but this can cause motor block^25^ and other local anaesthetic complications.

Postoperatively, patients with less pain can readily return to their normal activities. This is more crucial for mothers who wish to care for newborns. Our results revealed that patients in the ESPB group were consistently more satisfied. Decreased VAS values exhibited by patients in the ESPB group are a crucial factor in enhancing patient satisfaction. While the ESPB group exhibited lower VAS scores than the TAPB group, Boules et al.^15^ found no statistically significant difference in patient satisfaction between the two groups. In a study by Shukla et al.^26^ comparing bilateral ESPB and TAPB in patients undergoing hysterectomy, the ESPB group reported higher satisfaction. Patient satisfaction is a complex phenomenon that is affected by many parameters. As mothers’ satisfaction increases, their milk production rises, and it becomes better for them to breastfeed and care for their infants.

Because ESPB can be easily performed with USG guidance, it can be a viable alternative when neuraxial anaesthesia cannot be administered, such as in cases of vertebral anomalies or coagulopathy.^27^ Patients with a history of nausea and vomiting because of opioids used for postsurgical pain treatment can be candidates for ESPB.^28^ A patient who experienced severe post-CS pain after spinal anaesthesia wore off was successfully treated with an ESPB rescue block. The patient’s pain score decreased after 20 min, and no additional analgesics were required for approximately 12 h.^29^ Adding adjuvant drugs to local anaesthetics can prolong the block duration.

Peripheral nerve block techniques are beneficial in reducing post-CS pain, and these blocks have become an essential component of multimodal analgesia.^30^ Neuraxial anaesthesia is frequently preferred for CS. Compared with morphine, intrathecal fentanyl also provides intraoperative analgesia. However, its short duration of action necessitates the administration of additional analgesics during the postoperative period. Regional techniques, combined with acetaminophen and nonsteroidal anti-inflammatory drugs, ensure that the patient may remain IV opioid-free post-CS pain, similar to our study.

### Study Limitations

Our research has a few limitations. First, the patients were not blinded. Second, because of the effects of spinal anaesthesia, it is possible that the sensory extent of ESPB was not accurately measured. ESPB can be performed preoperatively, allowing for a more accurate evaluation of sensory extent. We applied ESPB at the end of the surgery because pregnancy makes preoperative application challenging. Therefore, patients could experience the analgesic effects of ESPB for a longer duration when it is performed after surgery. Moreover, the sensory extent was evaluated simultaneously in one plane. The change in extent over time was not evaluated.

## Conclusion

Inadequate pain management following CS is detrimental to the mother and the infant’s quality of life. In this population, it is essential to minimize IV opioid use and schedule additional analgesic treatment as part of a multimodal analgesic treatment approach. In this regard, USG-guided bilateral ESPB administration with spinal anaesthesia and low-dose local anaesthetics in postoperative pain management is a reliable approach that reduces pain intensity and postoperative analgesic consumption and safely increases patient satisfaction.

## Ethics

**Ethics Committee Approval:** Ethics committee approval was received from the Ankara University Faculty of Medicine, Human Research Ethics Committee (date: 13.02.2020, approval no.: İ2-87-20) before the study execution.

**Informed Consent:** Written informed consent was obtained from all participants.

## Figures and Tables

**Table 1. Amount of Bupivacaine Adjusted for Patient’s Height and Weight (mL)9:** 

**Patient weight (kg)**	**Patient height (cm)**								
	**140**	**145**	**150**	**155**	**160**	**165**	**170**	**175**	**180**
50	1.5	1.7	1.8	1.9					
55	1.5	1.6	1.8	1.9	2.0				
60	1.4	1.6	1.7	1.8	2.0	2.1			
65	1.4	1.5	1.7	1.8	1.9	2.1	2.2		
70	1.3	1.5	1.6	1.8	1.9	2.0	2.2	2.3	
75		1.4	1.6	1.7	1.9	2.0	2.1	2.3	2.4
80		1.4	1.5	1.7	1.8	2.0	2.1	2.2	2.4
85			1.5	1.6	1.8	1.9	2.1	2.2	2.3
90			1.4	1.6	1.7	1.9	2.0	2.2	2.3
95				1.5	1.7	1.8	2.0	2.1	2.3
100				1.5	1.7	1.8	1.9	2.1	2.2
105					1.6	1.7	1.9	2.0	2.2
110						1.7	1.8	2.0	2.2

**Table 2. Patients’ Demographics and Intraoperative Variables:** 

		**Group​**		
		**ESPB ​(n = 49)**	**Control (n = 50)​**	**p-value​**
**Age​ (year)**	Mean ± SD	30.41±5.58​	30.82±5.07​	0.701​^a^
**BMI​ (kg m^-2^)**	Mean ± SD	28.96±3.85​	30.08±3.24​	0.118​^a^
**Gestational age (week)​**	Mean ± SD	38.69±1.50​	38.72±0.88​	0.903​^a^
**Bupivacaine (mg)​**	Mean ± SD	9.58±0.76​	9.64±0.74​	0.699​^a^
**T4 time (min)**	Mean ± SD	6.78±1.76​	6.24±2.61​	0.094​^b^
**Bromage score at T4 time** 1 2 3	n (%) n (%) n (%)	0 (0) 21 (42.9) 28 (57.1)	1 (2.0) 25 (50.0) 24 (48.0)	0.482^c^
**Hypotension**	n (%)	28 (57.1)	35 (70.0)	0.184^c^
**Ephedrine requirement**	n (%)	28 (57.1)	35 (70.0)	0.184^c^
**Bradycardia**	n (%)	6 (12.2)	5 (10.0)	0.722^c^
**Atropine requirement**	n (%)	6 (12.2)	5 (10.0)	0.722^c^
**Surgery time (min.)​**	Mean ± SD	44.37±13.79​	38.24±13.04​	0.014​^b^
**First mobilization time (min.)​**	Mean ± SD	441.35±83.60​	426.68±85.7​	0.391​^a^
**Length of hospital stay (hour)​**	Mean ± SD	46.16±17.30​	51.12±21.86​	0.484​^b^

^a^Student’s t-test, ^b^Mann-Whitney U test, ^c^Chi-square test.

p<0.05 is taken as statistically significant.

SD, standard deviation; BMI, body mass index; ESPB, erector spinae plane block.

**Table 3. VAS Scores and Satisfaction Values:** 

**Time**		**Group**		
		**ESPB ​(n=49)**	**Control (n=50)​**	**p-value**
**2^nd ^hour**	VAS rest	0.81±1.39	2.84±2.48	<0.001^a^
	VAS movement	0.84±1.45	3.44±2.70	<0.001^a^
	VAS cough	0.88±1.51	3.64±2.78	<0.001^a^
	Satisfaction	0.88±1.15	2.36±2.15	<0.001^a^
**4^th ^hour**	VAS rest	2.37±1.48	4.08±2.40	<0.001^a^
	VAS movement	2.45±1.53	5.00±2.31	<0.001^a^
	VAS cough	2.39±1.50	5.02±2.51	<0.001^a^
	Satisfaction	1.00±1.22	2.60±2.31	<0.001^a^
**6^th^ hour**	VAS rest	2.98±1.53	4.30±2.41	0.002^a^
	VAS movement	3.53±1.68	5.22±2.39	<0.001^a^
	VAS cough	3.53±1.79	5.40±2.48	<0.001^a^
	Satisfaction	1.10±1.50	2.66±2.52	0.001^a^
**12^th ^hour**	VAS rest	2.71±1.80	3.78±2.49	0.025^a^
	VAS movement	3.12±1.83	4.76±2.57	0.001^a^
	VAS cough	2.98±2.04	4.92±2.73	<0.001^a^
	Satisfaction	1.08±1.72	2.38±2.28	0.001^a^
**24^th^ hour**	VAS rest	2.04±1.83	1.98±1.83	0.828^a^
	VAS movement	2.49±1.85	2.92±1.68	0.180^a^
	VAS cough	2.31±1.95	3.08±2.06	0.053^a^
	Satisfaction	0.69±1.06	1.54±1.67	0.003^a^

^a^Mann-Whitney U test. Values are presented as mean ± standard deviation.

p<0.05 is taken as statistically significant.

VAS, visual analogue scale; ESPB, erector spinae plane block.
